# On the Pin-Bearing Strength of Additively Manufactured Polymer Parts

**DOI:** 10.3390/polym15071660

**Published:** 2023-03-27

**Authors:** Mohammad Reza Khosravani, Hadi Sadeghian, Majid R. Ayatollahi, Tamara Reinicke

**Affiliations:** 1 Chair of Product Development, University of Siegen, Paul-Bonatz-Str. 9-11, 57068 Siegen, Germany; 2 Fatigue and Fracture Research Laboratory, Center of Excellence in Experimental Solid Mechanics and Dynamics, School of Mechanical Engineering, Iran University of Science and Technology, Narmak, Tehran 16846, Iran

**Keywords:** additive manufacturing, pin-bearing, fracture, mechanical strength, SEM, DIC

## Abstract

Due to the wide scope of applications of additive manufacturing (AM) in making final products, the mechanical strength of AM parts has become very important. Therefore, different tests are being developed to determine the structural integrity of three-dimensional printed components. In this respect, the pin-bearing test is designed to evaluate the response of a fastener, plate, and hole to stress. In this study, two different polymer materials were used to fabricate the samples utilizing the fused deposition modeling technique. Since the specimen width and hole diameter have effects on the pin-bearing strength and structural integrity of the parts, we prepared the specimens with four hole diameters to determine the influence of this ratio. A series of tensile tests were performed, and the stiffness and pin-bearing strength of additively manufactured specimens were determined. The preferred bearing failure mode was observed in several tested specimens. Subsequently, a scanning electron microscope investigation was conducted on the damaged area of the examined specimens to obtain insights into the damage mechanisms and failure behavior of the aforementioned specimens. We used digital image correlation technique to determine the strain field of dumbbell-shaped test coupons. The results of this research can be utilized for new designs of AM parts with a higher mechanical strength.

## 1. Introduction

Although the fabrication of geometrically complex parts is not possible by conventional manufacturing processes, additive manufacturing (AM) techniques have been used for this purpose. In fact, AM differs from the traditional methods of fabrication, which changes the conventional removal procedure to an additional process [1]. AM, commonly known as three-dimensional (3D) printing or rapid prototyping, is a fabrication process based on layer-by-layer deposition of material [2]. The capabilities of fabricating complex geometries, with high sustainability, customization of different products, consolidation of sophisticated assemblies, and minimal material waste can be considered as benefits of rapid prototyping compared to traditional manufacturing techniques. Due to these advantages, 3D printing has become very popular in many fields, such as construction [3], the automotive industry [4], medicine [5], polymer composites [6], tooling and moulding industries [7], electronics [8], and the food industry [9]. There are various types of 3D printing processes, and all of them have their targeted applications. According to ASTM F2792-12 [10], 3D printing has been categorized into seven methods, in which material extrusion is used in this research work.

Since 3D printing shows a rapid growth of development, different engineering issues such as printing accuracy [11], fatigue strength [12], interfacial bonding [13], life time [14], mechanical properties [15], fracture behavior [16], impact strength [17], and buckling [18] have been investigated in this field. Three-dimensional printing was devised for fabrication of prototypes, but its application has recently changed to fabrication of final products. Consequently, the structural performance of 3D-printed parts has become very important. Considering the wide applications of 3D printing and the introduction of this technique in different industries, new investigations are required to study the mechanical strength of 3D-printed components. In this context, the mechanical strength and structural integrity of additively manufactured parts were investigated in existing studies [19,20,21,22]. For instance, in [23] tensile and flexural behavior of the structures fabricated by fused deposition modeling (FDM) were investigated. To this aim, solid and porous continuous carbon fiber-reinforced polymer composites with different infill density levels were printed and subjected to the tensile and flexural tests. The results indicated an increase in tensile strength and flexural strength with an increase in the fiber content. At the same time, in [24] the bonding strength of 3D-printed suture joint was studied. In this context, different interfaces were printed and tensile tests were performed. The experimental findings indicated that with the flat interface, the material in the interfacial layer was subjected to simple shear, but in the wavy interface, the material was subjected to complicated mixed mode load. Later, effects of printing speed on the mechanical behavior of printed structural elements were investigated in [25]. In detail, the test coupons were fabricated with various printing speeds using acrylonitrile butadiene styrene (ABS) and polylactic acid (PLA) materials. After a series of tensile and three-point bending tests, it was concluded that the specimens printed with the lowest speed, showed lowest elongation at failure, but exhibited the highest tensile strength. Recently, we investigated the fracture response of anisotropic 3D-printed components [26]. Indeed, the structural integrity and the fracture behavior of PLA and PLA-wood additively manufactured parts were studied. The obtained results confirmed that using 40% wood fibers decreases the mechanical strength. Although extant research works have been investigated the mechanical behavior of 3D-printed components, this issue still remains as a significant problem that requires resolution. In this respect, examining the mechanical strength of 3D-printed structural elements under pin-bearing conditions is beneficial towards obtaining a better understanding of failure mechanisms of additively manufactured parts.

In the current study, a series of bearing tests was performed to determine the maximum fracture loads and failure modes of 3D-printed polymer parts. To this aim, PLA and ABS materials were used and all test coupons were printed using the FDM process. As the ratio of the specimen width (*W*) to the hole diameter (*D*) have effects on the pin-bearing strength and structural integrity of the parts, the specimens were fabricated with two hole diameters to determine the influence of this ratio. In the experimental practices, we used the digital image correlation technique (DIC) to measure strain on the surface of the 3D-printed specimens. The remainder of this paper is structured as follows: in Section 2, the details of specimen preparation and experimental investigations are described. Verification of experiments by DIC are explained in Section 3. The documented results are explained and discussed in Section 4. Finally, a short summary is presented in Section 5.

## 2. Experimental Procedure

### 2.1. Specimen Preparation

In this study, two groups of test coupons were fabricated and examined under tensile test conditions: (i) dumbbell-shaped samples, and (ii) double-shear specimens. The first group of samples were examined to determine the basic mechanical properties of additively manufactured parts. In detail, the specimens were designed in a CAD platform according to Type I in ASTM D638 [27]. The saved “.stl” file was used to print dumbbell-shaped samples utilizing PLA and ABS materials, based on the FDM technique. The printing parameters are listed in Table 1.

**Figure 1:**
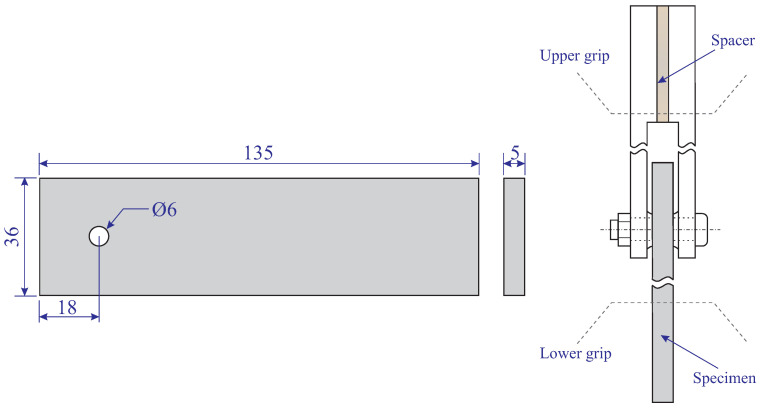
The schematics of double-shear samples and the fixture loading plate (dimensions in mm).

We designed and printed double-shear specimens for pin-bearing tests according to ASTM D5961-13 [28]. The double-shear specimen is a flat and constant rectangular cross-section specimen with a centerline hole near to the end of the specimen. The bearing load typically applied by a lightly torqued fastener (or pin) that is responded in a double shear through a fixture described in ASTM D5961-13 [28]. In the present study, the CAD platform was used to design double-shear samples. Subsequently, we imported the files in the Cura^TM^ slicing engine to slice and set the printing parameters. Finally, the file was used and the material was extruded and deposited in layers from a horizontal basis. The printed specimens were kept on the printer bed until they completely cooled down. This issue minimized the distortion and warping of the printed specimens. It is noteworthy that we fabricated a steel fixture according to its detail presented in ASTM D5961-13 [28] for experimental tests of double-shear samples. In the double-shear specimens, representative factors which have an influence on the failure mode are specimen width (*W*), hole diameter (*D*), and edge distance (*e*), which denotes the distance between the hole’s center and end of the specimen that is parallel to the force. Figure 1 demonstrates the schematics of double-shear samples and the fixture loading plate.

In the current research work, we designed, printed, and examined double-shear specimens with various W/D and e/D ratios. Indeed, the bearing failure mode might be attained by using high W/D and e/D ratios. As explained in [28], it is advised to consider the W/D and e/D ratios at least 6 and 3, respectively. Here, we fabricated specimens with diameter of 3, 4, 6, and 12 mm which give us different W/D and e/D ratios. Based on different hole diameters, the effects of hole diameter on the mechanical performance of the specimens can be determined. Since the specimen thickness has not a significant effect on the failure mode of the parts, all specimens were prepared with the same thickness.

### 2.2. Experimental Tests

**Figure 2:**
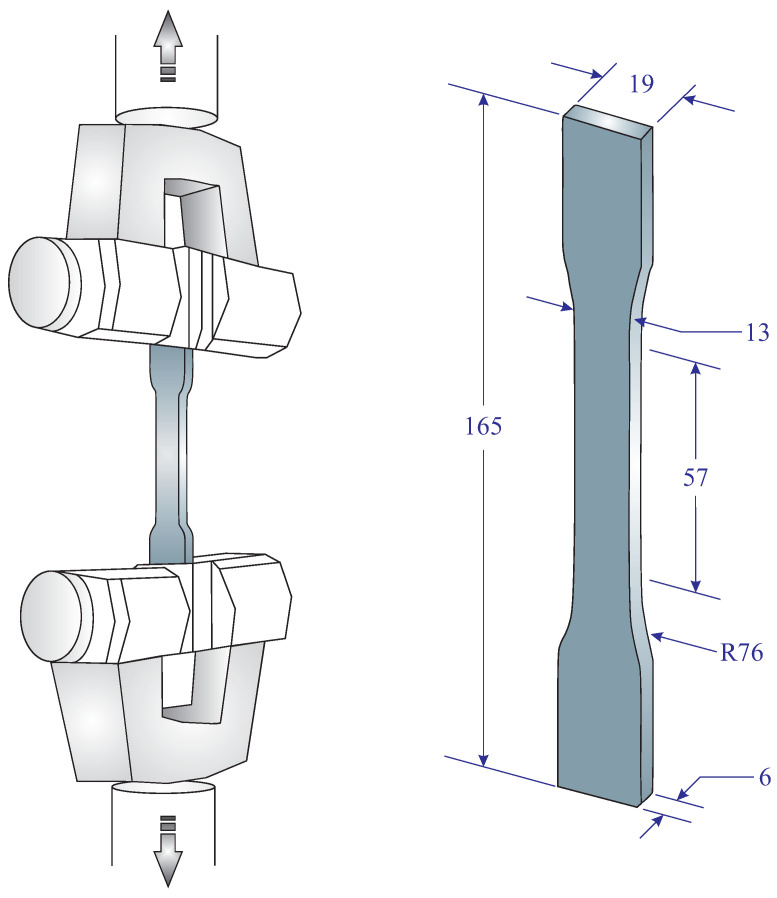
Geometries of a dumbbell-shaped specimen and schematic of tensile test conditions (dimensions in mm).

A series of tensile tests was conducted on the dumbbell-shaped samples. To this aim, we used SANTAM STM-150 uniaxial testing machine with a cross-head speed in the range of 0.001 mm/min to 500 mm/min equipped with a 50 kN load cell. The wedge grips of the machine directly gripped the shoulder parts of the specimens which were subjected to uniaxial tensile load. Appropriate tensile grips were used to keep the test coupons completely aligned in the vertical direction. In this study, the specimens experienced the constant crosshead speed of 5 mm/min until final failure. Geometries of a dumbbell-shaped specimen and tensile test conditions are schematically illustrated in Figure 2. In the present study, four identical dumbbell-shaped specimens were examined to ensure repeatability and reproducibility of the results.

**Figure 3:**
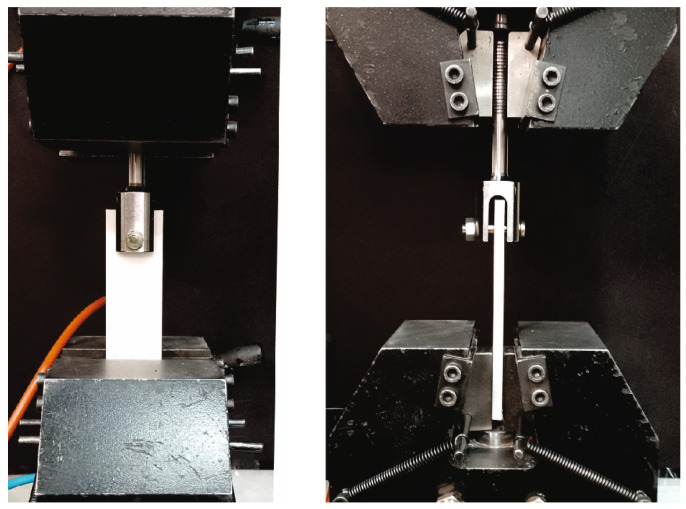
A 3D-printed specimen under test conditions, front view (**left**) and side view (**right**).

The tensile load was applied to all bearing samples. In this context, a tensile displacement with loading rate of 2 mm/min was applied. The experimental details are illustrated in Figure 3 where the top of the specimen was secured in displacement-controlled grips and the bottom of the specimen was secured in stationary grips. For repeatability of this experimental investigation, for each material and hole diameter, four identical specimens were printed and tested. Thus, thirty two tests were performed.

It has been discovered that performance of the the specimens is impacted by the clamping conditions. In fact, increasing the clamping pressure of a specimen lead to an increase in the bearing failure load. Therefore, utilizing an appropriate clamp is a necessity in experimental practice.

## 3. Verification of Experimental Tests

This section presents the application of the DIC technique for verification of our experiments. When a full field strain profile is required, DIC as a contactless and non-interferometric optical method provides a solution. DIC uses photogrammetric techniques to determine the full field and localized displacement and strain of an object. The determined displacement fields can be used to characterize the damage and rupture of the examined structural element [29]. In fact, by using the DIC technique, measurement quantities collected might be connected to the specific material reactions. DIC has been used extensively for small scale deformation events and strain measurements in different research works [30,31,32].

**Figure 4:**
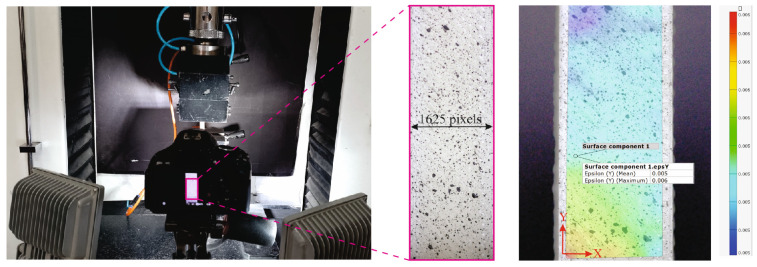
DIC setup including imaging and lighting equipment.

In the present study, the DIC technique is used to determine the strain field of dumbbell-shaped specimens. Since DIC is based on color contrast, we applied speckle patterns by spraying the specimen’s surface with contrasting paints. To this aim, a white spray paint was used to cover the specimen’s surface. Later, we sprayed spots of black mat paint, of random sizes on the white background. This technique provides a random texture that allows us to recognize the area around the point of interest and satisfies the contrast and randomness required for DIC measurement. Here, we used a Canon CMOS camera, equipped with Canon EF 100 mm macro lens supporting the spatial resolution equal to 2592 × 1728. Moreover, an appropriate illumination is provided by LED lamps. The utilized camera provided 30 frames per second for image acquisition. Figure 4 shows the DIC setup in our experiments.

In DIC measurement, the mm-to-pixel coefficient is used to indicate the scale of pictures. By measuring the width of the specimens in the pictures, it is discovered that the specimen width corresponds to 1625 pixels. The mm-to-pixel coefficient is calculated to be equal to 0.008 by dividing the specimen width (in mm) by its value in pixels. accordingly, 1 mm on the specimen equates to 125 pixels in the image. In our experimental practices, the step size and subset size for all DIC correlations were regarded to be 25 pixels and 45 × 45 pixels, respectively. As the test started, the first photo was captured. Afterwards, a snapshot of the deformed surface was taken every five seconds. The obtained results are presented and discussed in the subsequent section.

## 4. Results and Discussion

**Figure 5:**
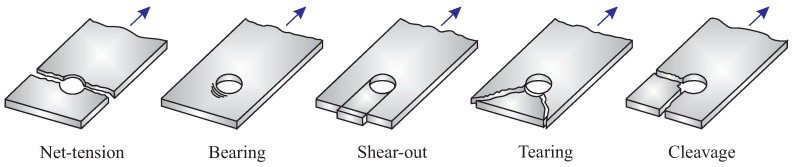
Schematics of different failure modes in bearing test.

In the bolted joints, the bearing failure mode is desirable, because it gives plenty of warning prior to the final failure, and develops slowly. In addition, the bearing failure mode is favorable, since load transfer capability can still be used. The tension, shear, and cleavage failure modes frequently occur abruptly and are typically catastrophic. Indeed, the term “net-tension” describes a situation wherein the joint capability for load transfer can no longer be sustained. Figure 5 shows typical failure modes which can be occurred in the bolted joints.

**Figure 6:**
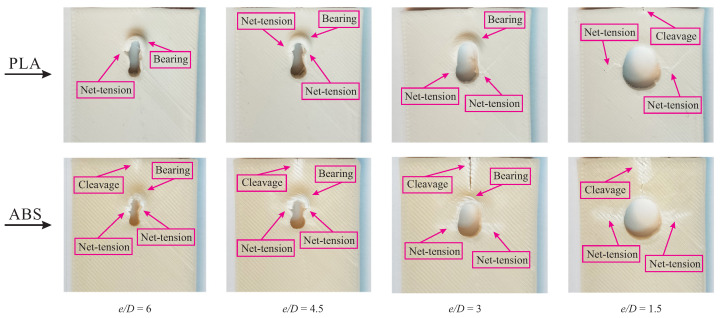
Examined specimens with various e/D ratio and failure modes.

In the present study, different failure modes are observed in the examined specimens. The failure mode can be determined by visual inspection and the geometry. Different failure modes obtained from experiments on PLA and ABS samples with various e/D ratios are illustrated in Figure 6. The hole deformation figures confirmed that pin-bearing corresponds to the first phenomena of material failure. As can be seen, neither PLA nor ABS specimens indicate the bearing failure mode in the test coupons with the maximum hole diameter (*D* = 12 mm). On the tested specimens, we documented the bearing failure mode for samples with e/D ratios ≥ 3.

According to the experiments performed on PLA and ABS test coupons, the failure loads of specimens with various W/D and e/D ratios are presented in Table 2. The results indicate that the highest failure load is 3646 ± 143 N belongs to a 3D-printed ABS samples with W/D=3 and e/D=1.5. Moreover, experimental findings confirmed that the lowest failure load was 2761 ± 90 N obtained from test on a PLA specimen while its W/D and e/D were 12 and 6, respectively. All specimens indicated a good replica repetition, and the maximum dispersion measured for failure load was less that 4%. In addition, the stiffness of specimens with different e/D and W/D ratios are presented in Table 2. Indeed, the highest stiffness is 1093 N/mm related to a 3D-printed ABS specimen.

In the current study, the ultimate bearing strength of the examined specimens was calculated according to ASTM D5961-13 [28], using the following equation:

(1) $$F^{\mathrm{bru}}=\frac{P^{\mathrm{Max}}}{k\times D\times h}$$

where $F^{\mathrm{bru}}$ denotes the ultimate bearing strength and $P^{\mathrm{Max}}$ indicates the maximum load prior to the failure. Moreover, *k* is load per hole factor, and *h* and *D* are specimen thickness and hole diameter, respectively. The average of obtained ultimate bearing strength of tested PLA and ABS specimens are summarized in Table 3. This ultimate bearing strength shows the maximum load capability of a bearing specimen with respect to the printing and loading direction. The result of experimental practice summarized in Table 3 provides a fair idea of how altering geometrical parameters affect bearing strength. We have obtained the highest ultimate bearing strength equal to 189 ± 5 from a test on a 3D-printed ABS part with the hole diameter of 3 mm.

**Figure 7:**
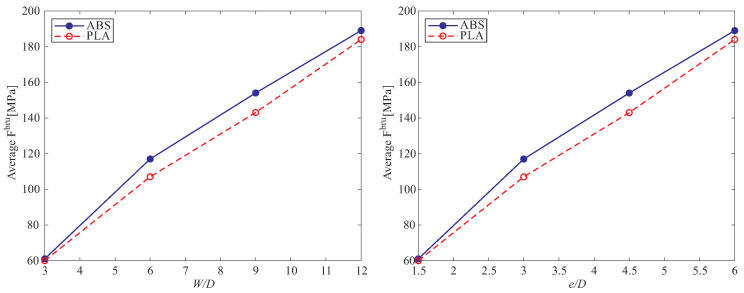
The ultimate bearing strength of tested specimens with various W/D and e/D ratio.

The ultimate bearing strength of tested PLA and ABS test coupons are illustrated in Figure 7. As is shown, the ultimate bearing strength increased with an increase in W/D and e/D ratio in both materials. In addition, the curves show that ABS has a higher ultimate bearing strength in all ratio of W/D and e/D compared to the PLA material.

**Figure 8:**
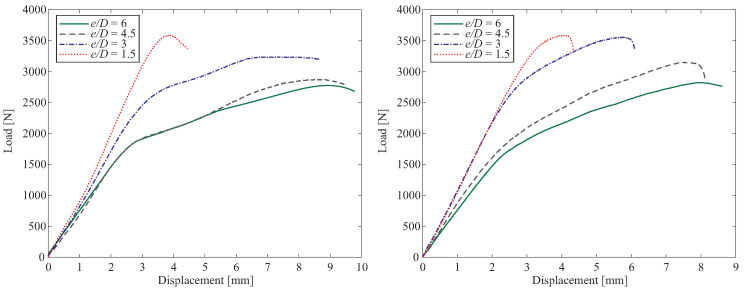
Load-displacement curves of examined PLA (**left**) and ABS (**right**) with different e/D ratios.

In Figure 8, the load-displacement results of tested PLA and ABS specimens with different e/D ratios are depicted. As illustrated, the highest loads belong to the lowest e/D ratio in both examined materials. In addition, the curves exhibit that the specimens with the smallest hole diameter (D=3) have the largest displacement in both PLA and ABS specimens. The illustrated curves in Figure 8 confirmed the crucial role of e/D ratio in determining maximum load capability of the specimens which is consequently efficient in leading the bearing failure mode.

**Figure 9:**
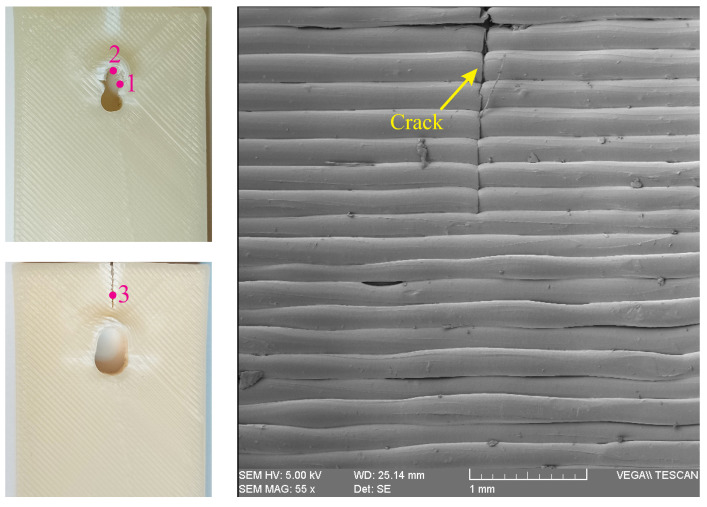
Reference points in visual inspection of specimens (**left**), and SEM image of point 1 of a 3D-printed ABS specimen with hole diameter of 12 mm (**right**).

In the current study, we used scanning electron microscopy (SEM) to provide a better insight into the fracture mechanism of the examined specimens. In this context, we have considered three points around the specimen hole (see Figure 9) to investigate the effects of loading on the bonds between filament layers. Point 1 is placed at the right side of the hole. Point 2 refers to the location where the pin was connected to the specimen and is located at the top side of the hole. Moreover, point 3 shows the fracture area, above the hole in a 3D-printed ABS specimen.

Figure 9 shows the SEM image of point 1 in a tested ABS specimen with hole diameter of 12 mm. This figure indicates that although the infill density was set to 100% in fabrication of the specimens, there are still some gaps between the filaments which confirm that these slight air gaps are an intrinsic feature of the FDM process. Moreover, this figure demonstrates a vertical crack that was created due to the net-tension failure.

**Figure 10:**
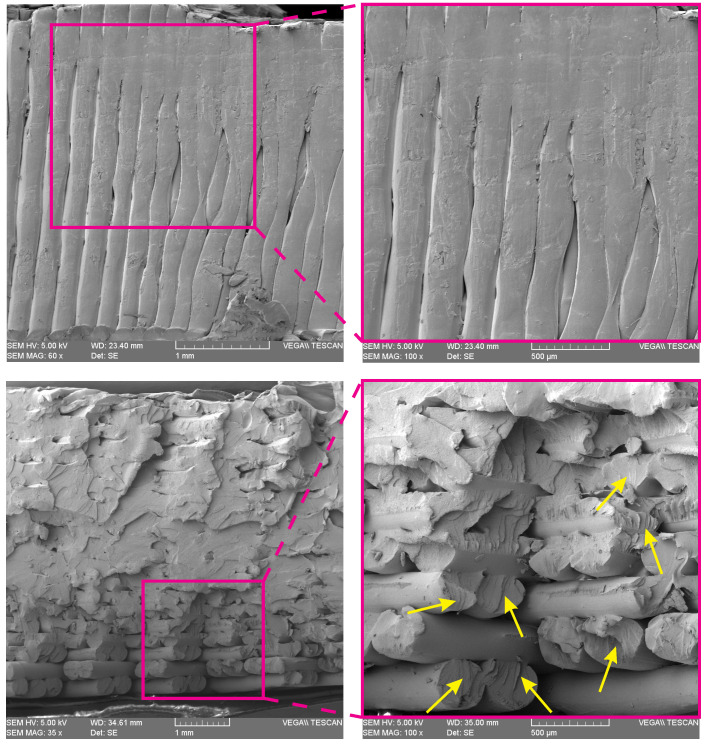
SEM images of point 2 (**top**) and point 3 (**bottom**) of a 3D-printed ABS specimen with hole diameter of 12 mm.

In Figure 10 the SEM images of two reference points (2 and 3) of a tested 3D-printed ABS specimen are illustrated. This specimen has the hole diameter of 12 mm (e/D=1.5) and shows net-tension and cleavage failure modes. In Figure 10 (top), there is a compression in the filaments due to the bearing load. The ridge marking pattern (yellow arrows) shown in Figure 10 (bottom) indicates that the rasters located at the top of the hole (point 3) tolerate a large portion of applied load where the inter-raster and inter-layer bonding strength bear a small portion of the applied stresses.

**Figure 11:**
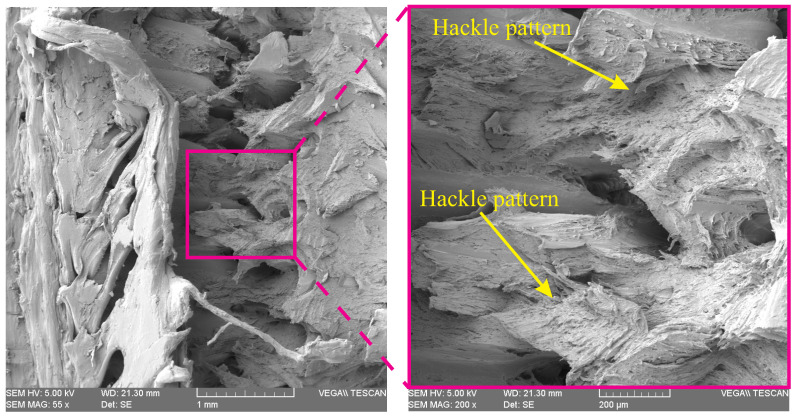
SEM images of point 1 of a 3D-printed PLA specimen with hole diameter of 3 mm.

Figure 11 demonstrates the fracture surface of point 1 of a 3D-printed PLA specimen with the hole diameter of 3 mm where the net-tension failure occurred. The hackle pattern shown in this figure implies the high energy dissipation resulting in large plastic deformation [33]. This issue is in accordance with the load-displacement curves shown in Figure 8 (left).

## 5. Conclusions

In the current study, the pin-bearing strength and the fracture behavior of additively manufactured polymeric components have been investigated. To this aim, PLA and ABS materials were used to fabricate double-shear specimens with four hole diameters to determine the influence of ratio of the specimen width to the hole diameter on the pin-bearing strength. It is noteworthy that different failure modes such as net-tension, bearing, and cleavage were observed in different specimens. Based on the results, the main contributions of this research are summarized as follows:

- The bearing failure mode was documented for test coupons with e/D ratios ≥ 3. The highest fracture load was 3646 ± 143 N belongs to a 3D-printed ABS specimen with W/D = 3 and e/D = 1.5.
- The highest ultimate bearing strength was equal to 189 ± 5 obtained from a test on a 3D-printed ABS part with the hole diameter of 3 mm.
- SEM investigations indicate that inter-raster and inter-layer bonding strength only bears a small part of the applied loads. However, the rasters at the top of the hole tolerate a substantial portion of the applied load.

Considering applications of 3D printing in fabrication of functional end-use products, the outcome of this research work is beneficial for future designs, development, and optimization of 3D-printed polymer components.

## Figures and Tables

**Table 1:** Processing parameters and properties of 3D-printed PLA and ABS specimens.

Parameters	PLA	ABS
Nozzle temperature (°C)	220	235
Bed temperature (°C)	60	100
Nozzle speed (mm/s)	60	60
Infill percentage (%)	100	100
Density (gr/cm^3^)	1.29	1.13
Layer width (mm)	0.42	0.42
Layer height (mm)	0.25	0.25
Number of contours	2	2
Raster angle (°)	±45	±45

**Table 2:** The results of the examined 3D-printed specimens.

Material	W/D	e/D	Failure Load (N)	Stiffness (N/mm)	Typical Failure Mode
PLA	3	1.5	3595 ± 123	989	Net-tension
	6	3	3218 ± 20	832	Bearing
	9	4.5	2850 ± 49	785	Bearing
	12	6	2761 ± 90	768	Bearing
ABS	3	1.5	3646 ± 143	1093	Cleavage
	6	3	3521 ± 52	1083	Cleavage
	9	4.5	3081 ± 73	843	Bearing
	12	6	2833 ± 72	769	Bearing

**Table 3:** The ultimate bearing strength of tested specimens.

Material	W/D	e/D	$F^{\mathrm{bru}}$ (MPa)
PLA	3	1.5	60 ± 2
	6	3	107 ± 1
	9	4.5	143 ± 2
	12	6	184 ± 6
ABS	3	1.5	61 ± 2
	6	3	117 ± 2
	9	4.5	154 ± 4
	12	6	189 ± 5

## Data Availability

The data presented in this study are available on request from the corresponding author.
